# Crystaline Gold. “J. T.” January, 1855

**Published:** 1856-01

**Authors:** 


### CRYSTALLINE GOLD.

[A "Review of Editorial Remarks upon Sponge Gold, by Dr. J. D. White," in "The Dental Register of the West." By "J. T." January, 1855.]

The following article from the *Dental News Letter*, we give our readers, first, because it is in part an answer to a review of an article of Dr. J. D. White, by J. T., which we published in the July number of the Register, and second, because we have the further explanation of Dr. White's theory of the contraction and expansion of gold in the teeth.

The doctrine advocated by Dr. White explaining the manner of the best of plugs dropping out, we must confess does not coincide with our views. We have always supposed that plugs dropped out from the fact that the cavities were not properly prepared, that is, were larger at the orifice than within, or that the gold had not been well compacted within. All filling in cavities not absolutely larger within than at the orifice according to the Doctor's theory, it appears to us, must come out, and particularly so if well put in.

We do not believe it necessary to serrate the walls of a cavity to make it hold a plug. Neither would we desire a deep cavity to be enlarged within, for this purpose, yet their are certain cavities in which we form the upper wall so as slightly to enlarge them at these points, these are particularly approximal civities difficult of access. Here the first blocks of gold are placed and this form of cavity, in the further introduction of the gold, and its consolidation rather draws the foil inward than otherwise, but even here the enlargement should be but trifling.

The amount of pressure requisite to consolidate a given amount of gold we have never tested, we always work, however, to make our plugs as solid as possible and are satisfied that Dr. White aims to do the same.

We can conceive it possible for dentine to absorb a certain amount of moisture, the amount would depend on its porosity. Would the enamel absorb any? The hardest of gold fillings in the mouth should resist the permeation of fluids. The exclusion of air and moisture is considered essential to arrest decay.

Does not the Doctor's reasoning lead us to infer that the gentleman of such "fine abilities" for filling teeth fail in many instances because he does his work too well. We would like to know if the young gentleman to whom he alludes and who has had his teeth "filled frequently" had not a further decay and softening of dentine manifested itself around the plugs before they were lost? Will not the walls of a cavity which are made up of inflamed dentine often undergo decomposition, even after a plug is well put in, while that portion directly covering the pulp organ may be restored to health?

We throw out these suggestions and ask these questions in all candor, and with an anxious desire to elicit every fact which may advance our Science, and do not pretend to discuss the question between the reviewer J. T. and Dr. White.

Dr. White hss given his objections fearlessly and candidly to sponge or crystal gold for filing teeth and they should be candidly and honestly met. If this is done, the profession must be benefitted by the discussion. ED.

We infer, from the language and style of the reviewer of J. T., that we have hit a tender point, but one that the majorrity of mankind is not free from—the *pocket.* Is there any "connection between the premises and the conclusions?" We never purposely set out to write nonsense, and if such matter should be found *abundantly* in our articles, an eqaually honest inquirer after truth will not waste much time and paper to prove it. If we were to compare notes with J. T., we could *guess* at the conclusion. We will therefore leave the *personal* consideration of J. T's. review, and proceed to the consideration of what we have said about sponge gold.

That packing gold into a cavity and rolling it into a plate are two things, is obvious; as a mass of sponge gold in rolling is not solid until the two surfaces are brought close together, and that the *amount* of pressure in the two operations is different. It is admitted that sponge gold can only be consolidated by "*pressure*," and that requires *muscular* strength, when it is not under the rollers. A plate of gold is not permeated by dampness'under ordinary pressure, but the hardest sponge gold plugs, made in the mouth, are. The hardest plugs of foil or sponge gold that we ever saw, are permeated by dampness to *some extent, more or less;* if not from the exposed surface, they are from the walls of the cavity. A tooth weighing 37 grains, lost five grains by drying, and took up four grains by immersing in water. Will this explain all we have said about that "*chap*" dampness? Go ask the school boy how this is effected, and he will answer by *capilliary attraction*, whether through dentine, between the folds of foil, or the crystals of sponge gold, or between the walls of the cavity and the plugs, or through relatively hard or soft plugs. This will explain the reason why sponge gold plugs have dropped out of their cavities; or any plug that has not a very strong hold on the walls of the cavity. As the sponge gold does not spread or crush latterly (as it is said) it is not as likely to get a firm hold on the walls of the cavity as foil; however this may also be an error. How does a mass of sponge gold acquire the form of plate, or wire, if it will not yield except directly before the instrument? This doubtless explains the difference between the pressure in *plugging* a tooth and *rolling* a plate.

We have said that we had occasion to remove plugs of this preparation, on account of the teeth giving trouble after they were plugged. We have also removed foil plugs for similar reasons. When a tooth gives trouble (pain) we remove the plug, because we have a better opportunity to treat the tooth, instead of leaving the case and the patient to take care of themselves; and it does not wound our conscience, but it affords us an opportunity to observe the character of the material used. We never attempted to plug a tooth with a mass of sponge gold sufficiently large to complete a filling, but use masses larger or smaller, as we had been directed by those who furnished the article; and we found that we could not render the plug hard enough not to be broken up in a short time by moisture, or some other cause. We used the gold as described by Dr. J. Taft, in an article which appears in the present number (Jan. 1855,) of the Dental Register, as faithfully as we knew how; we had been directed so to do before, by several in the profession, as the proper method. We did not, however, use it in as large portions as he directs, "as large as will freely enter the cavity, and then set in place with a large square pointed instrument, serrated upon the end;" and then follow with as small and as sharp a pointed instrument, and with as much pressure as the gold would bear without cutting it in pieces. We have said elsewhere that we had used the means that had been directed, and failed; and others in the profession have under similar circumstances, met with the same results. We have never said that a solid sponge gold plug is desolved by dampness. They are never made solid, at least we do not think so, if it is meant that solid means the same thing as complete welding. Nor do we believe, as it is used at present, that a plug can be made so in the mouth, either with sponge gold or foil; and when that is effected, it will be, in a measure, impervious to dampness; and sponge gold will be a good substance in some respects for plugging teeth; and then, as we have said, it will not *necessarily* be a *perfect plug*, if such a term is to mean that it is not liable to change by the vicissitudes of the mouth, and the changes the teeth undergo. We are doubtless the only dental author that does not seek after the vision—*perfection* in dental operation. If the plug is not also *welded* to the walls of the cavity, dampness will find its way between the plug and the tooth, and sooner or later destroy its use as a work of perfection.

We would be glad to know ourselves, why a very solid plug finds its way out of the cavity in the approximal and labial surface of teeth, or to be found starting from the cavity, as though it were a piece of gold plate, and that took firm hold of the cavity when the operation was performed, and the cavity dry, if it were not for the dampness *dissecting* (a new term) the gold away from the walls of the cavity. We will cite a case or two bearing upon this point.

A lady, about 30 years of age, applied to us to plug a back tooth, out of which the plug had fallen. She had been under the care of a gentleman of the finest abilities in our city for plugging teeth. All her front teeth had been plugged for some time. In examining them we remarked to her, that the plug of the left superior laterial, right approximal surface was coming out, and she must let us look at it from time to time. She called a few months after, to have some other operations done, when we observed that the plug had started out of the cavity still more; it was projecting out as much as the thickness of common card paper. We made an appointment, four weeks off, to refill the tooth, but in two weeks after, she called and informed us that the plug was gone. We examined the cavity, and found it was as well shaped as we usually make them for plugging. What forced this plug out? There are a number of plugs in the same mouth in similar progress of coming out.

We have a young gentleman under our care, from the same operator, whose teeth are all decayed near and on the necks, on the libial and buccal surfaces, and they came out by similar process; and they have been filled frequently; some of those plugs project so far out of the cavities, that six months ago we took a cast of them, in plaster, to preserve the phenomenon. The teeth of these patients are large. As these cavities hold plugs well enough to bear filling and burnishing without becoming loose at the time of plugging, why do they in time loosen?

We do not cite these cases as militating against the merits of sponge gold, but to show that there is some law, or laws, operating to dislodge plugs that have escaped "goggle-eyed" observation. We will give our *rationale,* or theory, and hold it good until a better is furnished. It is safe to begin by saying, that all substances expand by an increase of temperature, and contract by diminution of the same; and also that metals are good conductors of caloric, and this explains, in part, the reason why a tooth is sensitive to cold substances, when cleansed of the poor conductor—the decayed dentine. A small plug in a tooth will frequently be sufficient, when cold is applied to it, to excite or induce considerable pain, that before the plug was put in was insensible to such change; the surface of the whole body of the tooth was not sufficient from its non-conducting or feebly conducting powers of temperature to produce the same amount of pain. We have not, nor do we regard it as essential to make experiments to prove the "*exact*" difference between the expansion of tooth substance and a metal like gold; even if the ultimate difference were equal, it would make no difference in the argument or statement, since the gold is the more rapidly expanded or contracted. There has been too much stress put upon the gold adhering together and forming a solid mass, and by that means obtaining a "*perfect*" plug; and not sufficient upon the shape of the cavity to hold the mass against any other causes; and when sponge gold was discovered to "weld," and foil would not, it was hailed as the *desideratum; dampness, expansion and contraction* was not dreamed of.

But, to our *rationale.* It has too generally been considered that when a cavity was sufficiently enlarged within to hold or take a plug well, when the cavity was as dry as it could be made, and a little rough also, that it was properly prepared. But this is not so; a very illy-shaped cavity will take a plug, and hold it well for the time being, which the least effect of dampness or shifting by contraction or expansion, will let go its hold of the plug and causes it to drop out. All cavities ought to be larger within their orifices—a good deal, we do not know by actual measurement how much—and polished smoothly with pumice, before they are ready to receive a plug; and no considerable reliance to be placed upon the adhesiveness of the laminæ of the gold, *but upon the mechanical*

*hold the cavity takes of the whole mass of the plug;* adhesiveness only *favors* the *facility* of *manipulating* with the gold at the time of plugging, and makes it a LESS SKILLFUL performance than when this condition is not relied on. We make this *old-fashioned* statement with *fear* and *trembling*, and expect the whole *phalanx* of advocates of *adhesive* materials down on us, with an *adhesiveness* and *tenacity* of contact that we will not be able to shake off. This is our conviction, and we leave it to "time! the corrector, where our judgements err—the test of truth."

Mr. J. T. inquires, "does it not depend more upon the skill of the operator, and the perfect form of his instruments, than upon muscular strength? We answer, that depends as much upon the *adaptation* of instruments—not "*perfect form*"—as muscular strength; but the one is as necessary as the other; as it is *only* pressure that consolidates sponge gold or foil, a proper combination of both is requisite; and a stronger operator can make proportionately a better plug with the same instruments, as he applies more pressure than a weak one.

He further adds, "imagine a strong robust man filling a delicate lady's tooth with a force of from one to two hundred pounds; (!!) you could apply more force than that, could you not?" We must confess, we do not think we could apply so much force as J. T., if he is in the habit of applying from one to two hundred pounds. We may further add, that we have seldom seen so much exaggerated nonsense in an article of the same length, as is contained in the review of J. T. We will give the history of an experiment which we made before the class, which may throw some light on this statement of J. T. We have invented a simple instrument for registering the amount of pressure employed in consolidating gold foil in a tooth, out of the mouth, a description of which we will give hereafter, with a number of experiments, in connection with Prof. Arthur, for any one's use.

The experiment was made by plugging a cavity that held about three grains of gold; the smallest amount of pressure worthy of notice, at each effort with the plugger, was ten pounds, and the highest made, was forty-eight pounds. Pressure was made ranging between these two points, until the plug was considered as solid as it was ever made in the mouth, if not more so. When the aggregate was summed up, it amounted to two thousand three hundred and forty-two pounds. The tooth was broken, and the plug removed in a mass, and comparing it with other plugs taken from teeth plugged in the mouth, it was much harder than any we have ever seen, and absorbs dampness less rapidly than any we have tried. We consider that we applied as much pressure on this plug, and as *skillfully*, as we ever do in the mouth on a plug of the same size, and yet it does not come up to the enormous estimate of J. T. We question whether we are as strong as J. T. thinks we are ?

We have not space to reply as we would wish to the article of Mr. Rice, which we give in the present number, but we would thank him for the compliments he has been pleased to pay us, and the anxiety he manifested of endeavoring to arrive at the truth, and would, also, suggest, that he will make more rapid progress in gaining the desired object by support. ing his own statements, than by distorting our statements to his own ends and discrediting the correctness of our observations, which were made with equally as honest motives as his own, and that we had a better opportunity of observation in our cases than he. But to the consideration of the merits of the materials, sponge gold, as a suitable substance for plugging teeth.

We do not admit that we ever took a position against sponge gold, nor did we invite discussion; we invariably asked for more facts and information, not opinions, and said that we had made an honest endeavor to accomplish what had been claimed for it by others, and had used the means and methods recommended by its advocates, and had failed to make satisfactory operations, and asked for further instructions; we had a right as a journalist to express our doubts about it, or be liable to be regarded as favoring it; as we admitted articles in the journal strongly advocating its use, in the great majority of cases, still we do not make this statement to shield us in any way from any attack or review or our own remarks upon it. When the truth is known, and sponge gold is proven to be better than foil, in "important respects," we will doubtless have the courage to say so. Sponge gold was offered to the profession as a superior substance for plugging teeth by one who was not himself a dentist, and it was left for the members of the profession to say, whether it would meet their wants better than foil, and we offer the following reasons and considerations, why it will not meet our wants, and regard them as answering much that has been said in its favor, and in explanation of some things that we have said on this subject: gold in its crystallized state, as well as other metals that take that form is too hard to be made very compact by pressure, without frequent annealing, and we are compelled to obtain *in the tooth*, by pressure alone, in using sponge gold,—the density of the substance of the gold, what is done for us in preparing foil by melting, rolling and hammering, *out of the tooth;* pressure, in other words, will not render the substance of the gold as compact as melting and hammering. Therefore,

1st. The coarser preparation of sponge gold is too hard and brittle, and requires too much pressure to condense it.

2d. The finer preparation is too loose and bulky, and requires too much time to pack it into so small a bulk as a plug, and the preparations are always varying between these two extremes.

3d. It is unhandy, as it can only be used with the plyer or rough ended instrument.

4th. It deteriorates too much by transportation, retention or exposure, and handling while in use, and frequent annealing, to keep it in condition, finally spoils it.

5th. It is injuriously affected by the dampness of the mouth, whether the saliva is in actual contact with it or not.

6th. There is no certainty when a good plug is made with it, and it may look like a good plug when it is not.

7th. It requires, according to its advocates, *too* much *skill* to use it *properly.*

8th. A lack of perfection in its preparation, in any requisite, renders it entirely useless.

9th. It cannot be applied with facility in the majority of cases.

10th. Its *apparent* "welding" properties renders it deceptive as a substance for plugging.

11th. We do not know when it is in a proper condition for use, until we entirely fail with it.

12th. The merits claimed for it as a good substance for plugging, seem to depend upon such small circumstances or conditions, as to render it extremely uncertain or doubtful whether it is ever in a proper condition for successful use, and moreover, the term *welding*, as used in urging its use as a superior substance for plugging is calculated to deceive the operator, as it is incorrect, in speaking of the union of the particles of gold in plugging a tooth. The property of welding being confined to a very limited number of metals, of which gold is not one, welding also requires a temperature approaching that of fusion, to cause the union of the particles, and pressure also, and can take place under no other conditions, it is therefore, absurd to urge that property of metals to support it as a reliable form of gold for our purposes. The union of sponge gold in a plug is simply due to the close contact of the particles under pressure, and is an instance of simple *cohesion*, like that existing between the freshly cut surfaces of a leaden bullet when brought in contact under pressure. When sponge gold is compressed into a small cavity it is almost impossible, by any degree of force whatever, to bring the particles so closely together that the resulting mass should be free from pores, although it may appear solid to the eye. It is true the same difficulty exists, but in a much less degree in gold foil, *it* already being perfectly united in *one* direction, i. e. *laterally*, therefore, when compressed into a plug opposing its transverse section to the continuance of pores and fissures. The most important property, however, distinguishing sponge gold from gold foil, is that of the property possessed by many substances when in the form of a spongy porous mass, or fine powder, of condensing within their cavities many times their bulk of various gases, particularly oxygen. This has not yet, to our knowledge, been taken into account in considering the subject of sponge gold, and doubtless explains many of its peculiar and varying conditions heretofore unexplained, and perhaps unthought of. It is well known that platinum possesses this power to an astonishing extent, causing the instantaneous inflammation of hydrogen gas, when a jet of it is directed upon a lump of spongy platinum in atmospheric air. Gold, also, posseses this curious property even when in the form of foil; still more so, when in the spongy state, although, under all conditions, in a less degree than platinum. According to Dulong and Thenard, (Gmelin's Chemic. band., v. i, p. 509, (produces the ignition of a mixture of hydrogen and oxygen gases, when it is in the state of fine foil, at a temperature of 260° c. (=500° F.) Coarser foil, at 280° c. (=536° F.) Precipitated gold dust produces the same phenomena, at a temperature of 55° c. (=131° F.) In a more finely divided state, at 50° c. (=122° F.) Consequently, we may assume that gold possesses a greater power of condensing gases upon its surface in the form of sponge gold, than when in the state of foil, and helps to explain its extreme liability to deterioration. As it is evidently impossible to expel the gases perfectly from the porous mass, when it is converted into a plug, inasmuch as it is impossible to destroy its porous condition by mechanical pressure in the mouth, although the plug may outwardly appear to the eye solid, hence the brick-dust formation in the body of a plug. It needs no further argument to prove that the tooth is not in a condition to be preserved from further change, since the oxygen contained within the spongy mass will exert its chemical action upon the parts of the tooth with which it is in contact. This will doubtless explain, also, the blackening of the tooth when plugged with sponge gold. The same thing is observed also, in some teeth when plugged with certain specimens of foil. Thus examined, we consider the preparations of sponge gold as unworthy of the high considerations claimed for it by its advocates, and added to it, the remaining impurities of the metals, with which it is combined, in its preparation, especially mercury, which has so strong an affinity for gold, and superadded the influence of capillary attraction when exposed to dampness, upon so porous a mass as sponge gold, together with the heat of the mouth, it may contain, therefore, and we believe it does contain, the "*elements of its own destruction.*" J. D. W.

